# Outpaced by Drought: Weak Local Adaptation and Limited Potential for Drought Adaptation in a Deciduous Conifer

**DOI:** 10.1111/eva.70291

**Published:** 2026-07-18

**Authors:** Beth A. Roskilly, Martin R. Henry, Brandon M. Lind, Sally N. Aitken

**Affiliations:** ^1^ Forest and Conservation Sciences University of British Columbia Vancouver British Columbia Canada; ^2^ USDA‐Forest Service, Pacific Northwest Research Station Corvallis Oregon USA; ^3^ Department of Ecology and Evolutionary Biology University of Toronto Toronto Ontario Canada; ^4^ Department of Ecology & Evolutionary Biology, and Institute for Systems Genomics University of Connecticut Storrs Connecticut USA

## Abstract

As drought impacts on forests intensify, evaluating the potential for adaptation is critical for predicting forest responses and the impacts of management strategies. We assessed phenotypic and genomic variation in western larch ( *Larix occidentalis* Nutt.) populations to evaluate the potential for drought adaptation across its range. First, we established a seedling common garden experiment outside the natural range with 52 populations subjected to two drought treatments. Second, we analyzed pooled targeted exome‐sequence capture data from 44 populations of an adult provenance trial to evaluate the relative ability of climate, geography, and neutral genetic structure to predict landscape genomic variation. We found that population differentiation for drought resistance was low. Drought reduced population differentiation for growth and bud set, and weakened clinal associations for growth. We found no antagonistic correlations between drought resistance and growth or phenology, indicating that trade‐offs are unlikely to constrain adaptation. Further, landscape genomic variation was primarily explained by neutral genetic structure, with little additional variation explained by climate or geography. Together, these findings demonstrate weak local adaptation to drought and reduced genetic variation in traits under drought indicate that natural populations of western larch may have limited potential to adapt to future drought conditions.

## 1 Introduction

As global temperatures rise and droughts become more frequent, more severe, and longer, forests are becoming increasingly vulnerable to extreme weather events and biotic agents of injury (Allen et al. 2015; Anderegg et al. 2020; Hartmann et al. 2022). To persist, tree populations will need to respond to rapidly shifting climatic conditions with a combination of migration, phenotypic plasticity, and adaptation to new environments (Aitken et al. 2008). Due to their long generation times, tree populations have limited capacity to adapt or migrate quickly enough to track the rapid pace of change, increasing the risk of maladaptation to future drought. Evaluating the spatial scale and strength of local adaptation to drought and whether tree populations possess sufficient genetic variation to adapt to ongoing changes in drought conditions is necessary to predict the potential for adaptation, risks of maladaptation, and the potential impacts of mitigation strategies such as assisted migration.

The strength and scale of local adaptation to drought, as well as the amount and distribution of genetic variation in drought tolerance, is far from well resolved in trees (Moran et al. 2017). Trees commonly exhibit local adaptation to climate, and temperature is the primary driver, sometimes followed by precipitation, in most temperate and boreal species (Alberto et al. 2013; Leites and Benito Garzón 2023). Genetic variation in drought resistance traits and evidence for local adaptation can be assessed using common garden experiments, which allow for the partitioning of phenotypic variance into genetic and environmental components (Alberto et al. 2013). Local adaptation evolves under divergent selection and is characterized by populations that have higher fitness in their home environments relative to away environments, and when local populations perform better than foreign populations (Hereford 2009; Kawecki and Ebert 2004). Phenotypic divergence among populations and genetic clines with source environments found in common garden environments can also reflect local adaptation (De Villemereuil et al. 2016; Kooyers et al. 2015; Savolainen et al. 2013).

Studying adaptation to drought in trees poses unique challenges. First, it is difficult to study genetic variation in phenotypic traits relevant to drought adaptation, in part because of the complex responses trees possess to cope with drought and the various functional traits that may be involved (Moran et al. 2017). Second, drought responses may vary depending on drought severity and duration, or the spatial heterogeneity of soil moisture (Moran et al. 2017; Plomion et al. 2016). Common garden studies of seedlings allow for more controlled drought conditions, which can be used to assess genetic variation in traits relevant to drought tolerance that are difficult to measure in adult trees in the field. For example, declines in dark‐adapted chlorophyll fluorescence (F_v_/F_m_) can be rapidly assessed in seedlings to track reductions in photosynthetic efficiency in response to drought (e.g., Candido‐Ribeiro and Aitken 2024). Fluorescence declines are a late‐stage response to drought stress (Garcia‐Forner et al. 2015; Trueba et al. 2019) and can therefore be an informative trait reflecting drought resistance, or the ability to grow and survive under drought, a component of drought tolerance.

Landscape genomic studies provide a complementary approach to common garden studies to identify the genomic signal of local adaptation, its spatial patterns and environmental drivers (Exposito‐Alonso et al. 2018; Fitzpatrick and Keller 2015; Sork et al. 2013; Wadgymar et al. 2017). For example, a few landscape genomic studies in pine species have detected signals of local adaptation associated with soil water availability using genotype‐environment associations (GEA; Lind et al. 2017; Scotti et al. 2023; Budde et al. 2024). Genotype‐environment associations have been used to identify climatic variables associated with candidate loci in *Pinus contorta*, which agreed strongly with the variables associated with height growth from 20‐year‐old provenance trials, and to a lesser extent, seedling common garden phenotypes (Mahony et al. 2019). Landscape genomic studies also have the potential to estimate current and project future maladaptation to climates with genomic offsets (Capblancq et al. 2020). However, one of the major challenges to characterizing the genomic basis of adaptation is disentangling adaptive from confounding non‐adaptive factors underlying landscape genomic structure, such as isolation by distance and postglacial recolonization along environmental gradients (Hoban et al. 2016). Determining whether GEA signals are attributable to local adaptation to climate or confounded by geography or demographic history is therefore important and can be done by variance partitioning of landscape genomic variation with multivariate methods such as redundancy analyses (Capblancq and Forester 2021).

Genetic variation is the basis for evolution by natural selection, and thus the amount of genetic variation that is expressed under changing stressful climatic conditions is a key component of a population's adaptive potential (Fisher 1930; Shaw and Shaw 2014). Substantial genetic variation exists in natural populations of tree species (Alberto et al. 2013; Howe et al. 2003), but stressful environmental conditions can affect the amount of genetic variation expressed in adaptive traits (Bemmels and Anderson 2019; Hoffmann and Merilä 1999). Stressful conditions could reduce genetic variance in adaptive traits (Bemmels and Anderson 2019; Heblack et al. 2024) or cryptic genetic variance could emerge under stress to facilitate adaptation (Ledon‐Rettig et al. 2014; McGuigan and Sgrò 2009). The ability of populations to adapt to future drought will depend on the quantitative genetic variation expressed in relevant traits under drought conditions. Common garden studies that manipulate soil moisture allow for the estimates of quantitative genetic variation under conditions that may resemble future drought stress (Torres‐Martínez et al. 2019).

Even when substantial genetic variation in phenotypic traits is maintained under stressful conditions, genetic correlations among traits can constrain or facilitate adaptation (Agrawal and Stinchcombe 2009; Ahrens et al. 2024; Chevin 2013; Etterson and Shaw 2001). Temperate and boreal tree populations have evolved timing of active growth so that trade‐offs between growth and survival due to seasonal frost risks are optimized (Howe et al. 2003); however, genetic correlations among growth, phenology, and drought tolerance are less well resolved. In some conifer species, evidence of trade‐offs between growth and drought tolerance has been found among natural populations; trees from drier climates often exhibit more conservative growth strategies (De La Mata et al. 2014; Kerr et al. 2015; Cregg and Zhang 2001; Kolb et al. 2016; Matías et al. 2014). Bud phenology may also manifest in trade‐offs with drought tolerance. For example, early bud flush and bud set may also be associated with drought tolerance, as it could allow for increased early‐season growth and growth cessation before drought becomes limiting. In populations of *Pseudotsuga menziesii var. menziesii* (coastal Douglas‐fir), earlier bud flush has been associated with drier source climates and latitude, but earlier bud flush also increases the risk of late spring frost injury (Campbell and Sugano 1979; St Clair et al. 2005). Evaluating genetic correlations and potential trade‐offs among growth, phenology, and drought tolerance traits is therefore important for predictions of adaptation to future drought.

Here we studied the relationships among phenotypic and genomic variation, geography, and climate in natural populations of the deciduous conifer species western larch ( *Larix occidentalis* ). We established a seedling common garden experiment with a range‐wide collection of 52 natural populations. Two drought treatments were imposed of varying duration and severity relative to a well‐watered control treatment. We measured height growth, bud phenology, and drought resistance traits (chlorophyll fluorescence, canopy loss, and mortality) in the drought year, in addition to growth and bud break phenology in the following recovery year. Further, we evaluated the relative predictive power of climate, geography, and neutral genetic structure on landscape genomic variation to address the following hypotheses: (1) drought resistance traits exhibit population differentiation and clines consistent with local adaptation; (2) a significant proportion of landscape genomic variation can be attributed to climate, independent of geography and neutral genetic structure; (3) stressful drought conditions limit the amount of genetic variation expressed in adaptive traits; and (4) drought resistance traits are negatively correlated with growth and later bud phenology, indicating constraints on adaptive responses to drought.

## 2 Materials and Methods

### 2.1 Study Design

**FIGURE 1:**
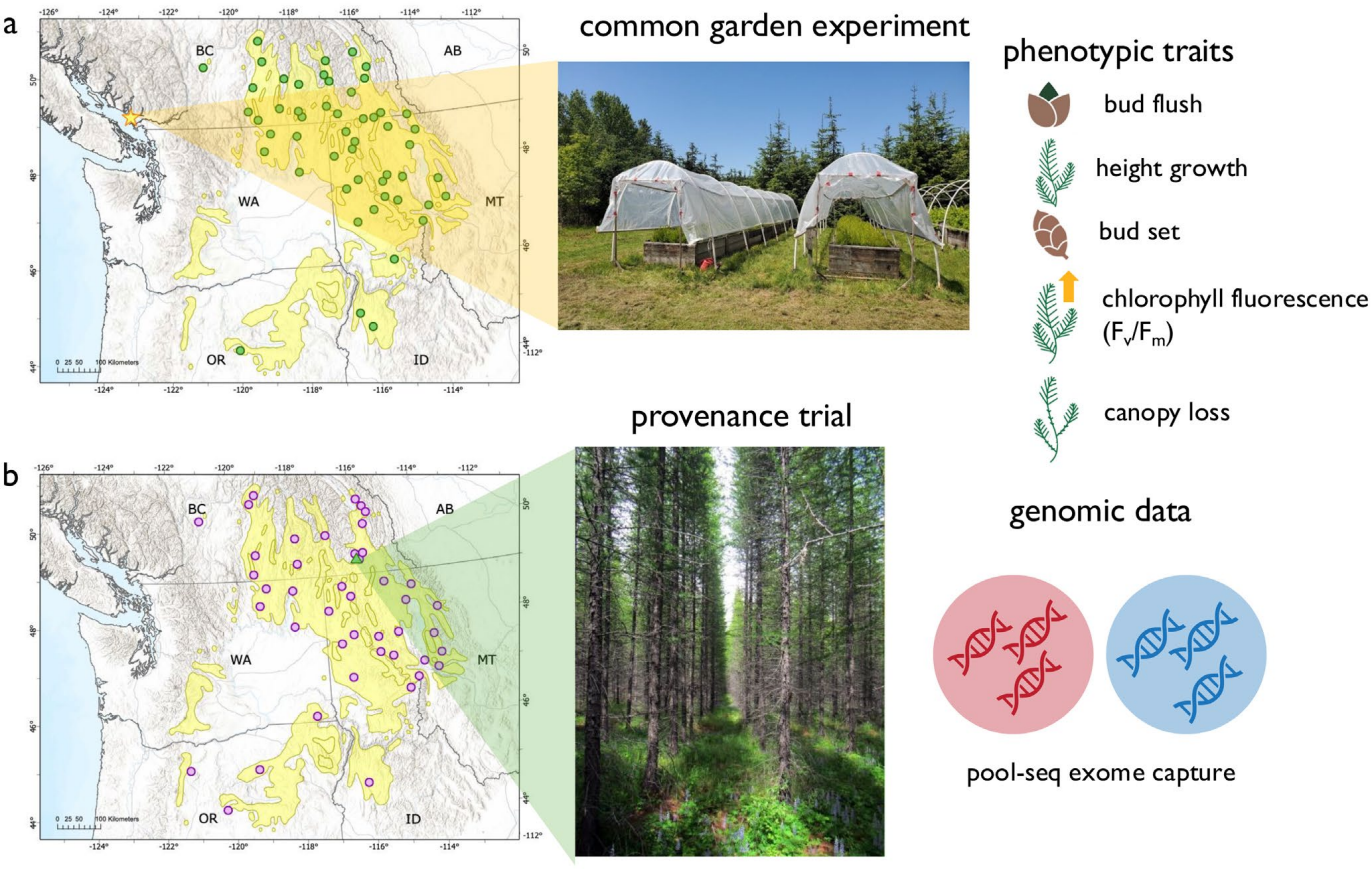
Phenotypic and genomic study design: (a) a common garden experiment (gold star) was planted with seed from 52 natural populations (green circles) shown on the distribution of the natural range of western larch (yellow polygon). Phenotypic traits were measured on plants in the common garden experiment; (b) a provenance trial in southeast British Columbia with 44 natural populations (purple circles) was sampled for genomic data (pool‐seq exome capture).

The seedling common garden study used for assessing phenotypic traits was described in detail in Roskilly and Aitken (2024) and is summarized here. It included 52 open‐pollinated seedlots from populations across most of the natural range (Figure 1a). We selected populations from available seedlots dispersed in geographic and climatic space, spanning a range of 6 degrees latitude, 7 degrees longitude, 1100 m elevation, 6°C mean annual temperature, and 1325 mm mean annual precipitation (Table S1). Seedlots were obtained from stored seed collections from the Rocky Mountain Research Station, Inland Empire Tree Improvement Cooperative, and British Columbia Tree Seed Centre. Each seedlot was bulked from at least 10 maternal trees in British Columbia and 5–10 maternal trees in the United States. Populations from the interior portion of the range (i.e., Rocky Mountain region) are well represented whereas populations from the westernmost portion of the range (i.e., Cascades region) were not included in the study due to inadequate samples in available collections.

For the genomic data, we sampled populations from the Lamb Creek provenance trial planted in southeast BC in 1991 (Rehfeldt and Jaquish 2010; Figure 1). This trial included 128 populations represented by seven seedlings planted in row plots with five blocks in a complete randomized block design. Foliage was collected from 39 of the 128 populations from the Lamb Creek trial and from five additional natural populations in the seedling common garden experiment. Populations were selected to span the geographic and climatic range of the species (Figure 1).

The common garden study design was described in detail in Roskilly and Aitken (2024). The experiment included a well‐watered control and two drought treatments. Thick sheets of polyethylene were stapled to the wood walls of the beds as a moisture barrier between treatments. Landscape cloth and a layer of gravel were placed at the bottom of the beds to hinder root penetration and prevent water moving up through capillary action from the ground soil below. We planted seedlings in four blocks in a complete randomized block design, with 12 plants per seed source per treatment, totaling 2880 seedlings in the entire experiment.

### 2.2 Drought Treatments

The drought treatments were described in detail in Roskilly et al. (2025), with a control, moderate, and severe drought treatment applied over the 2021 growing season (Figure S1). The summer of 2021 had a record‐breaking heat dome event in June and a prolonged drought in the Pacific Northwestern region of the United States and British Columbia (Thompson et al. 2022). Briefly, a mild drought pre‐acclimation phase was implemented in the drought treatments during the growing season preceding the final drought treatments in summer 2020. We suspended transparent rain covers over the beds on July 15th, 2020. We administered the full drought treatments in summer 2021 by suspending transparent rain covers over the beds on May 18th, 2021. The moderate drought treatment was not rewatered until August 13th, 2021, after almost 3 months without water, and the severe drought treatment was not rewatered until October 14th, 2021 (almost 5 months without water). The control treatment was regularly watered throughout the summers of 2020 and 2021.

### 2.3 Climate and Geographic Data

We used the climate normal period 1961–1990 for the geographic coordinates of each population from ClimateNA (Wang et al. 2016). This is the earliest period for reliable data from weather stations and was assumed to represent historical conditions likely driving current patterns of local adaptation. Five provenance annual climate variables were selected to test for associations: mean coldest month temperature (MCMT), continentality (TD) calculated as the difference between mean warmest month temperature and mean coldest month temperature, frost‐free period (FFP), mean annual precipitation (MAP), and summer‐heat moisture index (SHM). The geographic variables latitude, longitude, and elevation for provenances were also analyzed to test associations.

### 2.4 Phenotypic Data

We measured seven traits in the summer of the drought treatments: height growth, bud flush and bud set phenology, fluorescence (F_v_/F_m_) declines during drought, final fluorescence (F_v_/F_m_), canopy loss, and mortality, as described in detail in Roskilly et al. (2025). Height was measured five times during the 2021 growing season, with final height recorded on September 6th, 2021, after growth cessation and final bud set. Bud flush and early height growth were assessed during the recovery year (2022); early growth was calculated as the difference between heights measured on May 9th and July 7th, 2022. Data collected later in the recovery season were excluded due to reduced vigor and elevated mortality caused by competition from tight spacing.

Terminal bud flush and bud set were assessed visually as indicators of growth initiation and cessation. Bud flush was defined as the date when bud scales separated and needles became visible, and bud set as the appearance of brown bud scales on apical buds. Some control seedlings exhibited lammas growth, or reflushing after initial bud set, in which case final bud set was defined as the last date after which no reflushing occurred. Bud phenology was recorded weekly during spring flush (March 8th—April 13th 2021; March 11th—April 28th 2022), and bud set was monitored weekly from July 1st to September 6th, 2021. Phenological dates were analyzed as day of year.

To assess drought‐induced declines in photosynthetic efficiency, we measured dark‐adapted chlorophyll fluorescence (F_v_/F_m_) using a FluorPen Z990 fluorometer (Qubit Systems Inc.). Measurements were taken on mid‐canopy needles after ≥ 30 min of dark adaptation, achieved by covering beds with blackout and shade tarps near dusk. Fluorescence was measured seven times in drought treatments from June 16th to October 13th, 2021. All seedlings were measured initially, after which a reduced subset of control seedlings was assessed due to low variation. Measurement timing was guided by biweekly preliminary assessments of a subset of seedlings.

Canopy loss in drought‐treated seedlings was visually estimated on September 6th, 2021 and scored categorically (0%, 25%, 50%, 75%, or 100% foliage loss). Mortality was defined as the proportion of seedlings with no growth and exhibiting severe foliage loss by September 6th, 2021 and was confirmed the following season by absence of new growth on May 9th, 2022.

### 2.5 Genomic Data

Exome capture pooled‐sequencing data were generated for 20 individuals pooled per population. Exome capture probes were designed using high‐quality transcriptomic data targeting exonic regions of reference genomes (as in Lind et al. 2022). DNA was extracted from each individual tree and contributions equalized in DNA pools for each population. Pool‐seq libraries were sequenced in 150 bp paired‐end format on an Illumina HiSeq4000 instrument at the Centre d'expertise et de Services Génome Québec, Montréal, Canada. We mapped reads from western larch to an amended version of the current reference genome of coastal Douglas‐fir v1.01 (Neale et al. 2017). We used transcriptomic data from western larch and amended non‐mapping transcripts to the Douglas‐fir reference before mapping pool‐seq data. Adding non‐mapping transcripts ensured higher mapping rates of Illumina sequencing reads to the amended reference.

Single nucleotide polymorphisms (SNPs) were called using a bioinformatics pipeline (described in Lind et al. 2022), and filtered for 25% missing data across populations, minimum read depth per population per locus ≥ 8, and global minor allele frequency ≥ 0.05. Paralogs can lead to erroneous SNP calls due to misalignment to a reference genome (Rellstab et al. 2019). To avoid bioinformatic artefacts due to the presence of paralogs, SNPs were also filtered with the pipeline to exclude loci that were also called as heterozygous from haploid megagametophyte tissue (see Lind et al. 2022 for more details).

### 2.6 Analyses

#### 2.6.1 Treatment Effects

To test for the effects of treatment and account for genetic variation among populations for phenotypic traits, we used a linear mixed‐effect model using residual maximum likelihood with ASReml‐R version 4.0 (Butler et al. 2017). The following mixed‐effects model was used:

(1) $$Y_{\text{ijkl}}=\mu +P_{j}+T_{k}+{\left(P*T\right)}_{\mathrm{jk}}+B_{l}+\epsilon_{\text{ijkl}},$$

where $Y_{\text{ijkl}}$ is the phenotype corresponding to individual *i* from population *j*, treatment *k* and block *l*; μ is the phenotypic global mean across all individuals (fixed intercept); T is the fixed effect of treatment *k*; *p* is the coefficient for the random effect of the population *j*; P*T the random interaction effect of seedlot *j* and treatment *k*; *B* is the random effect of the *l*th block; and $\epsilon_{\text{ijkl}}$ is the random residual error term. To account for spatial effects on traits, row and column positions within the experiment were incorporated into the random residual term with a correlation structure. Models including the spatially correlated residual term consistently improved model fit and reduced AIC values. We tested for differences between the best linear unbiased estimators (BLUEs) of treatments using pairwise *t*‐tests with the *asremlPlus* package for ASReml‐R version 4.0 and adjusted *p*‐values using Tukey's method for multiple comparisons. To assess the significance of seedlot‐by‐treatment interactions for each trait, we conducted a likelihood ratio test using nested models with and without the seedlot × treatment random effect.

### 2.7 Estimating Drought Resistance

To quantify the rate of fluorescence decline over time as a measure of drought tolerance, F_v_/F_m_ values for each individual plant at a given date of measurement were first corrected for spatial autocorrelation based on an autoregressive model of residuals (Candido‐Ribeiro and Aitken 2024). A beta regression model (Ferrari and Cribari‐Neto 2004) with spatially‐corrected individual F_v_/F_m_ values was then used to estimate the linear rate of decline (slope) of fluorescence over time (date of measurement) using the R package *betareg* (Cribari‐Neto and Zeileis 2010). The slopes of fluorescence declines were then used in the mixed‐effects model represented by Equation (1) and the following models. When needed, we used a cubed root transformation of slope values (after adding a constant to make values positive) and a log transformation of F_v_/F_m_ values to meet model assumptions of normality of residuals and homoscedasticity of variances.

### 2.8 Population Differentiation, Phenotypic Clines, and Trait Correlations

To test for population differentiation in phenotypic traits for each treatment separately, we estimated population variance components for each trait and best linear unbiased predictions (BLUPs) of populations by fitting mixed‐effects models using a reduced version of Equation (1) with no treatment effects. We calculated two metrics: V_POP_, a measure of the amount of phenotypic variance among populations relative to the total phenotypic variance, and Q_ST_, a standardized measure of the amount of genetic variance among populations relative to the total genetic variance. V_POP_ is calculated as the phenotypic variance among populations divided by the sum of the residual variance and the variance among populations. V_POP_ is often considered as a proxy for Q_ST_ when relationships among individuals within populations are not known. However, it is an underestimation of Q_ST_ because in the denominator, the total phenotypic variance is used (i.e., population and residual component), rather than the sum of the among‐population component and twice the additive variance component used to calculate Q_ST_ (Liepe et al. 2016). For comparison, we approximated Q_ST_ as the phenotypic variance among populations divided by the sum of twice the additive variance component estimated from full‐sib families (described in Roskilly et al. 2025) and the phenotypic variance among populations. Q_ST_ estimates were then qualitatively compared with global F_ST_, a standardized measure of neutral genetic differentiation among populations, estimated with 1.4 million SNPs as a multilocus F_ST_ with the *poolfstat* R package (Gautier et al. 2022). Q_ST_ estimates greater than global F_ST_ were considered evidence of local adaptation for a given trait (Whitlock and Guillaume 2009).

In order to detect clines, we tested associations between BLUPs of population phenotypes and the five selected climate variables, latitude, longitude, and elevation using simple linear regressions in R version 4.2.0 (R Development Core Team 2022). Regression coefficients were significant at *p* < 0.05. To test for correlations among traits, BLUPs among population phenotypes were used to estimate Pearson correlation coefficients, significant at *p* < 0.05.

### 2.9 Partitioning of Landscape Genomic Variation

We used a partial redundancy analysis (pRDA) for partitioning genetic variance to investigate the effects of climate or isolation by environment (IBE), geography, or isolation by distance (IBD), and demographic history (neutral genetic structure) on the population allele frequencies of 419,315 SNPs with no missing data for 44 western larch populations (Capblancq and Forester 2021). To estimate the effect of neutral genetic structure, we used five proxies of neutral genetic structure based on population scores along five axes of a genetic PCA using a subset of 3669 random SNPs, pruned for linkage disequilibrium to prevent bias from regions of low recombination (Lind et al. 2024). First, we randomly selected one locus per genomic reference contig longer than 1 kb with no missing data across populations, a minimum allele depth (per locus per population) of 20, and a maximum allele depth of 1000. We then performed linkage disequilibrium pruning by removing one locus from any pair with a squared Pearson *r* > 0.2658, corresponding to the 99.5th percentile of the distribution of pairwise r values among loci. The same five annual climate variables were included in the pRDA as used for the phenotypic analysis, and the effect of geography was characterized by population latitude, longitude, and elevation.

## 3 Results

### 3.1 Drought Treatment Effects

Drought treatments had significant effects on growth, bud phenology, fluorescence (F_v_/F_m_), canopy loss and mortality. Moderate and severe drought treatments reduced annual growth by an average of 29% (7.49 cm) and 37% (9.53 cm), respectively, and advanced bud set by 5.2 and 6.5 days, respectively (Figure S2). Fluorescence (F_v_/F_m_) declined by 22% on average in the severe drought treatment (Figure S3) and mortality averaged 14.6% (Figure S4). Canopy loss in response to moderate and severe drought was 29% and 45%, respectively (Figure S5). Early growth in the recovery year following drought was reduced by an average of 7% (0.85 cm) in the moderate and 22% (2.61 cm) in the severe drought treatment relative to the control (Figure S6).

### 3.2 Population Differentiation and Phenotypic Clines

The proportion of phenotypic variance due to population differences (V_POP_) was generally low and varied among traits, ranging from 0.05 to 0.12 in the control, 0.07 to 0.11 in the moderate, and 0.001 to 0.10 in the severe drought treatment (Table 1). The proportion of additive genetic variance among populations (Q_ST_) varied widely among traits, ranging from 0.003 to 0.74 (Table 1), though some values were inflated due to the low additive genetic variation in traits estimated with full‐sib families in the drought treatments.

**TABLE 1:** Population differentiation for traits in each treatment. Proportion of phenotypic variance among populations (V_POP_), genetic variance among populations (Q_ST_), and range of estimated population phenotypes. Bud flush and bud set were analyzed as day of year (doy). Bud flush occurred prior to implementation of the moderate and severe drought treatments. Phenotypic variance components: *σ* ^2^_p_ = phenotypic variance among populations, *σ* ^2^_e_ = phenotypic variance within populations and the residual variance.

Trait	Control				Moderate				Severe			
	*σ* ^2^_p_	*σ* ^2^_e_	V_POP_	Q_ST_	*σ* ^2^_p_	*σ* ^2^_e_	V_POP_	Q_ST_	*σ* ^2^_p_	*σ* ^2^_e_	V_POP_	Q_ST_
2021 annual growth (cm)	6.43	46.79	0.12	0.23	2.05	27.65	0.07	0.14	1.38	32.06	0.04	0.13
2022 early growth (cm)	7.04	63.46	0.10	0.19	7.70	61.69	0.11	0.21	0.04	40.83	0.001	0.003
2021 bud flush (doy)	3.00	31.68	0.09	0.24	4.07	35.26	0.10	0.44	3.22	28.05	0.10	0.20
2022 bud flush (doy)	3.13	54.29	0.05	0.06	4.37	51.67	0.08	0.47	1.83	45.86	0.04	0.12
2021 bud set (doy)	12.67	106.49	0.11	0.13	3.40	46.77	0.07	0.19	3.75	44.83	0.08	0.58
Canopy loss (%)					42.51	581.48	0.07	0.74	26.63	634.48	0.04	0.35
Final fluorescence (F_v_/F_m_)									0.005	1.35	0.004	0.05
Fluorescence decline (ΔF_v_/F_m_ per day)									0.0001	0.004	0.02	0.24

**FIGURE 2:**
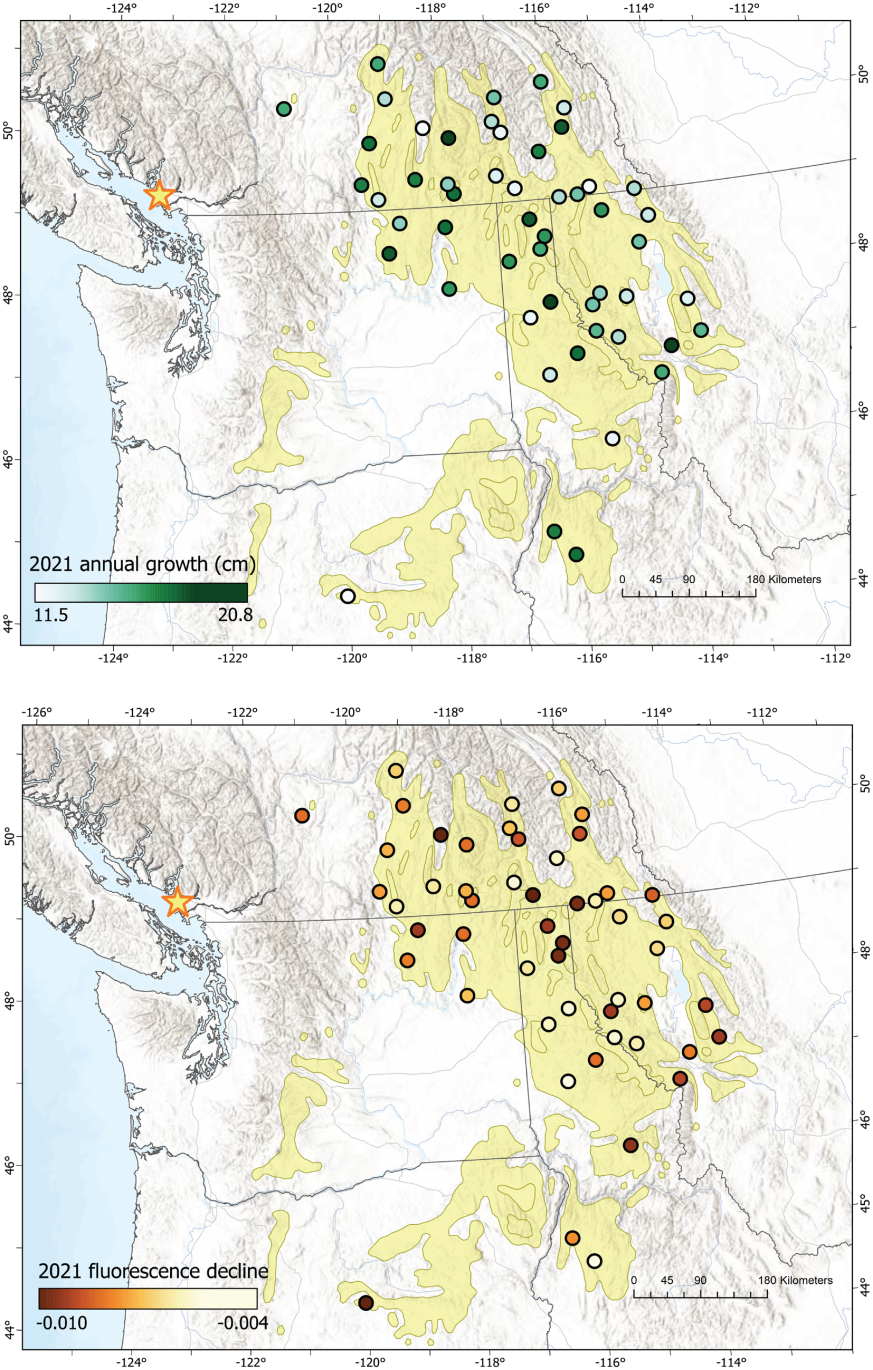
Maps showing the absence of geographic and climatic clines in growth and drought resistance (fluorescence declines) in the severe drought treatment. Color of points represent trait values (BLUPs) for annual growth (top) and fluorescence decline (ΔF_v_/F_m_ per day; bottom) in the severe drought treatment for 52 natural populations with the range of western larch in yellow and location of common garden test site (gold star) in western North America.

Drought resistance traits of final fluorescence and fluorescence declines in the severe drought treatment had low population differentiation (Table 1) and were not associated with any climatic or geographic variable (Table S2; Figure 2). Canopy loss had low population differentiation in the moderate (V_POP_ = 0.07; Table 1) and severe drought treatment (V_POP_ = 0.04; Table 1) and no clinal associations with geography or climate (Table S2).

Population differentiation for annual growth was moderately low in the control (V_POP_ = 0.12) and reduced by the drought treatments, with the lowest value in the severe drought treatment (V_POP_ = 0.04, Table 1). Annual growth in the drought year (2021) was weakly associated with longitude (*R* ^2^ = 0.12, *p* = 0.01) in the control treatment but was not associated with any climatic or geographic variable in the severe drought treatment (Figure 2; Table S2). The timing of bud set also had moderately low population differentiation (V_POP_ = 0.11) and was reduced by the drought treatments (V_POP_ = 0.07–0.08; Table 1). Bud set was weakly associated with elevation (*R* ^2^ = 0.15, *p* = 0.005) in the control treatment, and under severe drought, was associated with elevation (*R* ^2^ = 0.14, *p* = 0.005) and length of the frost‐free period (*R* ^2^ = 0.12, *p* = 0.01; Table 2). Population differentiation for bud flush timing in the drought year was low in the control (V_POP_ = 0.09) and similar in the drought treatments (Table 1). Bud flush in the drought year was weakly associated with longitude in the control treatment (*R* ^2^ = 0.16, *p* = 0.003) and in the severe drought treatment, associated with longitude (*R* ^*2*^ = 0.20, *p* < 0.001; Table 2) and SHM (*R* ^2^ = 0.10, *p* = 0.03; Table 2).

**TABLE 2:** Significant associations between phenotypic traits and geographic or climatic variable of source populations in the control and severe drought treatment. Coefficient of determination (*R* ^2^) and slope values at significance level of *p* < 0.05. FFP = frost‐free period length (days), SHM = summer heat moisture index, and ns = not significant. Drought resistance traits of fluorescence values, fluorescence declines, and canopy loss had no significant relationships with climate or geography.

Trait	Variable	Control		Severe drought	
		*R* ^2^	Slope	*R* ^2^	Slope
2021 annual growth (cm)	Longitude (°)	0.12	0.38	ns	ns
2021 bud flush (doy)	Longitude (°), SHM	0.16, ns	0.28, ns	0.20, 0.10	0.35, −0.02
2022 bud flush (doy)	Longitude (°), FFP (days)	0.38, 0.22	0.38, −0.02	0.08, ns	0.11, ns
2021 bud set (doy)	Elevation (m), FFP (days)	0.15, ns	−0.004, ns	0.14, 0.12	−0.002, 0.02

Early growth in the recovery year had moderately low population differentiation in both the control and moderate drought treatment (V_POP_ = 0.10, 0.11) and was substantially reduced in the severe drought treatment (V_POP_ = 0.001; Table 1). Population differentiation for bud flush in the recovery year (2022) was low in all treatments (V_POP_ = 0.04–0.08; Table 1). Bud flush in the recovery year was associated with longitude in the control (*R* ^2^ = 0.38, *p* < 0.001) and to a lesser extent in the severe drought treatment (*R* ^2^ = 0.08, *p* = 0.04), as well as FFP in the control treatment (*R* ^*2*^ = 0.22, *p* < 0.001).

### 3.3 Trait Correlations Among Populations

**FIGURE 3:**
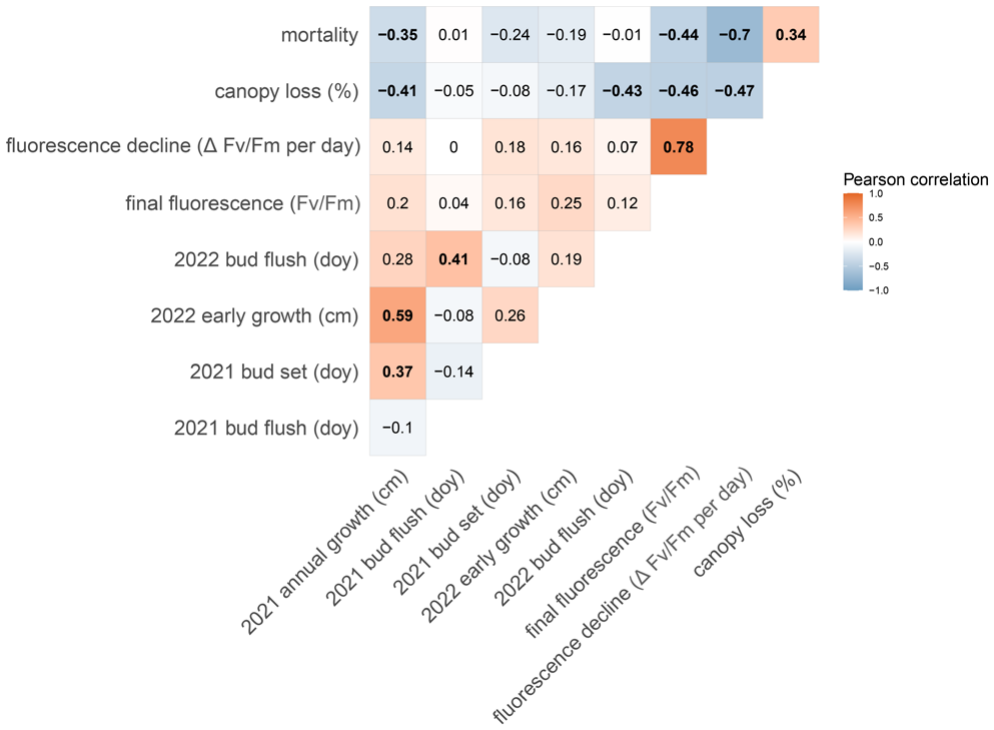
Trait correlations among population phenotypes (BLUPs) in the severe drought treatment in the drought year (2021) and recovery year (2022). Pearson correlation coefficients (*r*) significant at *p* < 0.05 are in bold.

Fluorescence declines, final fluorescence, and canopy loss in the severe drought treatment were all correlated with mortality among populations (Figure 3). The rate of fluorescence decline was strongly correlated with proportion of mortality (*r* = −0.7, *p* < 0.001) and percentage of canopy loss among natural populations (*r* = 0.34, *p* = 0.01). Annual growth in the drought year (2021) was negatively correlated with canopy loss (*r* = −0.41, *p* = 0.002) and proportion of mortality (*r* = −0.35, *p* = 0.01) but not correlated with final fluorescence or fluorescence declines. Bud flush and bud set phenology in the drought year were not correlated with drought resistance traits (Figure 3). Early growth in the recovery year was not correlated with drought resistance traits. Bud flush in the recovery year (2022) was earlier with greater canopy loss (*r* = −0.43, *p* = 0.002).

### 3.4 Partitioning of Landscape Genomic Variation

The full model with climate, neutral genetic structure, and geography explained 47% of the total genomic variance across western larch populations (Table 3). The pure effect of neutral genetic structure was highly significant and explained 27% of the total genetic variation (58% of the variance explained by the full model; Figure S7). The pure effect of climate explained 9% of the total genetic variation (20% of variance explained by the full model) and was not statistically significant. The pure effect of geography accounted for 6% of the total genetic variance (12% of the variance explained by the full model). A small proportion of genetic variance could not be attributed to any of the three sets of predictors (10% of explained variation), indicating a portion of variance is shared among predictors due to collinearity (Figure S8).

**TABLE 3:** The effects of climate, geography, and neutral genetic structure on genetic variation decomposed with partial redundancy analysis (pRDA). The proportion of explainable variance represents the total constrained variation explained by the full model.

	Variance	*R* ^2^	*p* (> F)	Proportion of explainable variance	Proportion of total variance
Full model: F~climate + geography + structure	285.11	0.47	0.001	1.00	0.47
Structure only: F~structure | (climate + geography)	165.86	0.27	0.001	0.58	0.27
Climate only: F~climate | (geography + structure)	55.87	0.09	0.242	0.20	0.09
Geography only: F~geography | (climate + structure)	34.33	0.06	0.196	0.12	0.06
Confounded structure/climate/geography	29.06			0.10	0.05
Total unexplained	322.12				0.53
Total variance	607.23				

## 4 Discussion

To evaluate the potential of populations to adapt to drought, we studied phenotypic and genomic variation in natural populations of western larch. Both phenotypic and genomic data showed weak evidence for local adaptation to climate generally and to drought specifically. Our results suggest that western larch populations may be globally rather than locally adapted to drought. Drought resistance traits showed no evidence of local adaptation, with low population differentiation and a lack of phenotypic clines with source environments. Population differentiation and clinal associations were weakened by drought for both growth and bud set timing in the severe drought treatment. The lack of antagonistic correlations between drought resistance traits and growth or phenology among populations suggests these relationships will not constrain adaptation to drought. Landscape genomic variation was primarily shaped by neutral genetic structure, with little additional variation explained by source climate or geography. Collectively, these findings signal weak local adaptation to drought and that adaptation may be limited by expressed genetic variation in traits under drought.

Weak evidence for local adaptation for western larch seedlings suggests that drought resistance could be a highly conserved trait. These findings are consistent with weak local adaptation for drought resistance found in seedlings of sympatric populations of *Pseudotsuga menziesii var. glauca* (interior Douglas‐fir) stressed to 74% mortality under greenhouse conditions (Candido‐Ribeiro and Aitken 2024). Further, evidence for population differentiation in embolism resistance, a trait strongly related to drought resistance, is limited in other Pinaceae species (Chauvin et al. 2019; Klein et al. 2013; Lamy et al. 2011). Embolism resistance and correlated drought resistance traits could be canalized as the targets of past natural selection rather than current divergent selection. Low genetic variation in drought resistance is consistent with classical life history theory, which predicts that traits closely related to fitness should be under strong directional selection and exhibit less genetic variance (Fisher 1930).

Growth and bud set phenology also lacked a distinct signal of local adaptation to aridity, though populations exhibited strong plastic responses (reduced growth and advanced bud set) to the drought treatments. Instead, weak clines in growth and bud set phenology were associated with geographic variables of longitude and elevation, respectively. The stronger associations of these traits with geographic variables over climate variables may indicate the integrated effect of multiple climatic selection pressures and demographic history. For example, across the range of western larch, longitude acts as a proxy for the transition from semi‐maritime climates of the Cascades to more continental climates of the interior, with harsher winters and distinct shifts in the seasonality of precipitation. Indeed, longitude was moderately correlated with the length of the frost‐free period, continentality, and summer heat moisture index (Figure S8). Longitude may also reflect the demographic history of post‐glacial recolonization. In this case, phenotypic variation in traits like growth could be the result of selection and migration coinciding along a longitudinal gradient, leading to a stronger association with a geographic variable than with interpolated climate variables.

Bud set was weakly correlated with elevation in both treatments and length of the frost‐free period in the drought treatment only. This pattern is consistent with many temperate conifers, where earlier bud set was found in higher elevation or higher latitude populations, reflecting an adaptation to avoid early fall frost risks in locations with shorter growing seasons (Alberto et al. 2013). Given that the study site was in a warmer climate than experienced within the natural range, an association with length of the frost‐free period could have been weakened in the well‐watered control treatment by the favorable conditions for late season lammas growth (reflushing after initial bud set), delaying final bud set.

Bud flush timing showed weak signals of local adaptation but a potentially adaptive plastic response to drought. Bud flush was weakly correlated with summer aridity, but only in response to mild drought exposure in the prior season. Bud flush was more strongly associated with longitude, i.e., eastern populations had later bud set, potentially an adaptation to avoid greater risk of late spring frosts. Interestingly, bud flush was substantially earlier at our warm study site compared to previous seedling trials within the natural range (G. E. Rehfeldt 1992), suggesting a strong plastic response to warm temperatures, with more rapid accumulation of forcing temperatures after chilling requirements are met. Earlier bud flush with exposure to warmer temperatures, a response seen across many tree species, could be an adaptive plastic response to increasing temperatures and summer droughts if it allows for faster early‐season growth before heat or drought become limiting.

Partitioning of landscape genomic variation did not reveal a distinct signal of local adaptation to climate, including aridity variables. We found that neutral genetic structure explained most of the genetic variance among populations. Climate and geography did not explain substantial additional genetic variance beyond that explained by population structure, indicating that demographic processes or gene flow barriers shape most of the explainable genetic variance among populations. This contrasts with strong genomic signals of local adaptation found in other co‐occurring widespread conifer species such as *Pinus contorta* and *Psuedotsuga menziesii* (Capblancq and Forester 2021; Lind et al. 2024; Mahony et al. 2019). While western larch has a relatively small geographic range for a conifer species, it spans 6°C for mean coldest month temperature and 1325 mm for mean annual precipitation; substantial climatic variation that could exert divergent selection on populations. Notably, we estimated low selectively neutral genetic differentiation among populations in this study (global F_ST_ = 0.01), indicating weak population structure. It is worth noting that the genomic signals detected here may have been biased due to use of the reference genome coastal Douglas‐fir v1.01 for aligning reads (Neale et al. 2017), although we were still able to detect over 1.4 million SNPs. The use of a high‐quality conspecific or congeneric reference genome may reveal different signals of landscape genomic structure (Akopyan et al. 2024). Nonetheless, our results indicate that high gene flow levels and demographic history may contribute to weak population structure and have diluted effects of divergent selection pressures due to climate.

Weak signals of local adaptation to drought at the range‐wide scale in this study are consistent with previous observations that local adaptation in most temperate and boreal conifer species is driven primarily by temperature and only secondarily by precipitation (Leites and Benito Garzón 2023). Seasonal temperature varies more predictably over broader spatial scales compared to precipitation and soil moisture, which could lead to more consistent selection pressures for temperature, facilitating local adaptation. However, studies in pine species have detected local adaptation over relatively fine spatial scales corresponding with soil water availability (Budde et al. 2024; Lind et al. 2017; Scotti et al. 2023). Our broad sampling of populations across the species range, rather than intensive sampling over topographically complex or edaphically variable regions of the range, limited our ability to assess genetic variation in drought resistance at finer spatial scales such as elevational gradients. Future studies could evaluate the spatial scale of adaptation to drought by sampling over fine as well as broader spatial scales.

Drought substantially reduced expressed genetic variation among populations in growth and bud set phenology, indicating a potential barrier to drought adaptation. These results are consistent with the reduced additive genetic variance and lower heritability we found among selectively‐bred families under the same drought treatments compared with the control treatment (Roskilly et al. 2025). Diminished genetic variation under drought has been found in other studies, such as in provenance growth responses of *Picea abies* in stressful marginal habitat (Klisz et al. 2019). A transplant study in the perennial forb *Boechera stricta* also found genetic variance and heritability are diminished when transplanted to drier low elevation environments (Bemmels and Anderson 2019). Further, a resurrection study in *Erythranthe cardinalis* found limited evidence for evolutionary responses to drought due to low heritability of traits related to drought adaptation (Sheth et al. 2026). Contrasting results have been found in some annual plants; for example, a strong evolutionary response in flowering time to drought was found in a resurrection study of *Brassica rapa* (Franks et al. 2007). In the annual *Lasthenia fremontii* that inhabits vernal pools in California, dry conditions can promote expression of genetic variation (Torres‐Martínez et al. 2019). In western larch seedlings, the reduced population differentiation and lower genetic variation in early growth we observed persisted into the recovery year in the severe drought but not in the moderate drought treatment, suggesting that moisture availability in late summer allowed for expression of genetic variation in early growth the following season. Nonetheless, the diminished genetic variation in growth and bud phenology under drought found in this study indicates potential limitations to drought adaptation.

Drought resistance traits were strongly intercorrelated, including a strong correlation between mortality and fluorescence declines, as found in previous studies (Garcia‐Forner et al. 2015; Roskilly et al. 2025). A strong association between fluorescence and mortality is consistent with fluorescence declines being a late‐stage signal of drought stress that occurs after plants have reached the point of hydraulic failure (Trueba et al. 2019). However, drought resistance of populations was not correlated with annual height growth or bud phenology. Other studies have found drought tolerance to be associated with slower growth among populations from contrasting climates in several conifer species (Bansal et al. 2015; Candido‐Ribeiro and Aitken 2024; De La Mata et al. 2014; Kerr et al. 2015; Montwé et al. 2015). Conservative growth strategies found in drought‐tolerant populations may also be associated with a suite of adaptive responses to colder winter temperatures, shorter growing seasons, greater continentality, and drier climates. Drought tolerance may be correlated with adaptation to colder climates because of overlapping morphological or physiological mechanisms that confer tolerance to freezing and winter desiccation (Anfodillo et al. 2002; Bansal et al. 2016; Mayr et al. 2006). Contrary to the strong signals of local adaptation in cold hardiness found in many temperate conifer species including subalpine larch (Alberto et al. 2013; Leites and Benito Garzón 2023; Vance et al. 2026), previous studies have found weak evidence for local adaptation to cold temperatures in western larch (G. E. Rehfeldt 1982; G. Rehfeldt 1995; Roskilly and Aitken 2024). Further, bud flush and bud set phenology were not correlated with drought resistance traits among populations. However, correlations with other unmeasured traits could constrain adaptation as climate change progresses (Bastias et al. 2024). Nonetheless, the lack of antagonistic correlations between drought resistance traits and growth or phenology indicates these relationships should not constrain adaptation to drought (Etterson and Shaw 2001).

Experimental drought treatments can be more controlled and evenly applied, and better monitored in common garden studies of seedlings, and physiological traits relevant to drought tolerance can be more easily phenotyped. However, the performance of seedlings may differ from those in the field or in older trees. Although we did not detect strong genetic differences in drought tolerance among populations in seedlings, they may exist in long‐lived tree species in the field or at later stages of development. Analyses of provenance trials in adult trees of several species have revealed significant population differentiation and clines in drought resilience, measured as the capacity to resume growth rates after a drought period using tree rings, for example *Pseudotsuga menziesii* , *Pinus contorta* , *Picea abies* , and *Picea glauca* (Depardieu et al. 2020; Montwé et al. 2015, 2016; Trujillo‐Moya et al. 2018). On the other hand, weak differences in drought resilience among populations have been detected in provenance trials of *Picea glauca, Pinus halepensis*, and *Pinus brutia* (Sang et al. 2019; Veuillen et al. 2023). These findings highlight that local adaptation in drought tolerance varies among species and can also be strongly affected by the intensity and duration of the drought events studied, the traits assessed, and the age of trees studied.

Weak local adaptation, low genetic variance in drought resistance traits, and reduced expression of genetic variation in growth and phenology traits under drought found in this study indicate potential limitations to drought adaptation for western larch populations. These barriers to drought adaptation are important considering that rising temperatures and intensifying droughts are likely to reduce climate suitability for western larch within its current range, leading to limited natural regeneration and recruitment (MacKenzie and Mahony 2021; Vieira et al. 2024). Planting drought‐hardy seedling stock may help mitigate problems with reforestation; for example, adaptive plastic responses could be used at the nursery stage to adjust traits like root plug size and root‐to‐shoot ratios to increase the chances of seedling establishment in the field (Grossnickle 2012; Pinto et al. 2023). Other silvicultural decisions such as microsite selection, site preparation, planting season, and density will also play an increasingly important role. However, the weak signals of local adaptation and potential limitations to drought adaptation found in this study suggest that species selection may become more important than seed source selection in areas of the range with decreasing climatic suitability due to hotter and drier conditions (MacKenzie and Mahony 2021). These findings, together with projected reductions in climatic suitability within the current range and expansion of suitable areas beyond (MacKenzie and Mahony 2021; Rehfeldt and Jaquish 2010), highlight that range expansion through assisted migration rather than assisted gene flow may be an important management strategy for western larch forests in the future.

## Funding

This work was supported by Genome Canada. Genome British Columbia. British Columbia Ministry of Forests. Forest Genetics Council of British Columbia. University of British Columbia. Natural Sciences and Engineering Research Council of Canada.

## Conflicts of Interest

The authors declare no conflicts of interest.

## Supporting information


**Figure S1:** Drought experiment timeline showing when rain covers were put over the raised bed (grey), when water was excluded (yellow) and when treatments were regularly watered (blue). Phenotypic traits as symbols show rough timing of measurements in 2021 and 2022 (drought and recovery season, respectively).
**Figure S2:** Annual growth and bud set phenology for the control, moderate, and severe drought treatments in western larch seedlings in the drought year (2021). Bold points represent the BLUEs for each treatment and lighter points represent individual data. Letters indicate statistically significant differences between groups (Tukey's HSD test, α = 0.05).
**Figure S3:** Fluorescence (F_v_/F_m_) values and decline curves over time in the control and severe drought treatment. Points and lines represent natural population phenotypes (BLUPs) and red‐orange color gradient represent fluorescence decline (slope) in the severe drought treatment. Fluorescence values in the control treatment are shown in green.
**Figure S4:** Proportion of mortality in the severe drought treatment by each natural population (seedlot). Orange bars represent proportion of seedlings that were dead by the end of the drought treatment and green represents proportion of seedlings that survived. Black line represents the average for all populations.
**Figure S5:** Canopy loss (%) in the moderate and severe drought treatment for natural populations. Bold points represent BLUEs of each treatment and lighter points represent individual data. Letters indicate statistically significant differences between groups (Tukey's HSD test, α = 0.05).
**Figure S6:** Early height growth (cm) by treatment in the recovery year (2022). Bold points represent BLUEs for each treatment and lighter points represent individual data. Letters indicate statistically significant differences between groups (Tukey's HSD test, α = 0.05).
**Figure S7:** Results of the partial RDA showing the loadings of each locus (SNPs; grey points), population allele frequencies (green points), and the predictor variables along the first two RDA axes (left) and the first and third axes (right). The locus scores (grey points) are rescaled to an unshown axis for better visibility.
**Figure S8:** Pearson correlations between pRDA predictor variables including the five selected climate variables, five neutral genetic PCs, latitude, longitude and elevation of each sampled population.
**Table S1:** Description of the 52 natural populations of Larix occidentalis and their phenotypes.
**Table S2:** Regression coefficients (R^2^) between phenotypic traits and geographic and climatic variables of the source location for each natural population.

**Data S1:** 2026_EVA_SI.

## Data Availability

The phenotypic data used in this study are publicly available at the Dryad Digital Repository: https://doi.org/10.5061/dryad.w3r22817g. The genomic sequence data (FASTQ files) generated for the *Larix occidentalis* samples in this study have been deposited in the National Center for Biotechnology Information (NCBI) Sequence Read Archive (SRA) under BioProject accession number PRJNA1434029. Individual BioSample metadata and attributes are available under accessions SAMN56354590 – SAMN56354633. All data will be made publicly available upon publication of this manuscript.
