# Supplementary material for: Outpaced by Drought: Weak Local Adaptation and Limited Potential for Drought Adaptation in a Deciduous Conifer
Source: Evol Appl. 2026 Jul 18;19(7):e70291. doi: 10.1111/eva.70291 (PMC13379777; doi:10.1111/eva.70291)
Supplement: Supplementary file 1 — Figure S1: Drought experiment timeline showing when rain covers were put over the raised bed (grey), when water was excluded (yellow) and when treatments were regularly watered (blue). Phenotypic traits as symbols show rough timing of measurements in 2021 and 2022 (drought and recovery season, respectively). Figure S2: Annual growth and bud set phenology for the control, moderate, and severe drought treatments in western larch seedlings in the drought year (2021). Bold points represent the BLUEs for each treatment and lighter points represent individual data. Letters indicate statistically significant differences between groups (Tukey's HSD test, α = 0.05). Figure S3: Fluorescence (Fv/Fm) values and decline curves over time in the control and severe drought treatment. Points and lines represent natural population phenotypes (BLUPs) and red‐orange color gradient represent fluorescence decline (slope) in the severe drought treatment. Fluorescence values in the control treatment are shown in green. Figure S4: Proportion of mortality in the severe drought treatment by each natural population (seedlot). Orange bars represent proportion of seedlings that were dead by the end of the drought treatment and green represents proportion of seedlings that survived. Black line represents the average for all populations. Figure S5: Canopy loss (%) in the moderate and severe drought treatment for natural populations. Bold points represent BLUEs of each treatment and lighter points represent individual data. Letters indicate statistically significant differences between groups (Tukey's HSD test, α = 0.05). Figure S6: Early height growth (cm) by treatment in the recovery year (2022). Bold points represent BLUEs for each treatment and lighter points represent individual data. Letters indicate statistically significant differences between groups (Tukey's HSD test, α = 0.05). Figure S7: Results of the partial RDA showing the loadings of each locus (SNPs; grey points), population allele [file EVA-19-e70291-s002.docx]

## Supplementary materials

Article title: **Outpaced by drought: weak local adaptation and limited potential for drought adaptation in a deciduous conifer**

The following supplementary materials are available for this article:

**Fig. S1** Drought experiment timeline

**Fig. S2** Annual growth and bud set phenology for the control, moderate, and severe drought treatments in western larch seedlings in the drought year (2021)

**Fig. S3** Fluorescence (Fv/Fm) values and decline curves over time in the control and severe drought treatment.

**Fig. S4** Proportion of mortality in the severe drought treatment by each natural population

**Fig. S5** Canopy loss (%) in the moderate and severe drought treatment for natural populations.

**Fig. S6** Early height growth (cm) by treatment in the recovery year (2022).

**Fig. S7** Results of the partial RDA showing the loadings of each locus (SNPs), population allele frequencies, and the predictor variables.

**Fig. S8** Pearson correlations between pRDA predictor variables including the five selected climate variables, five neutral genetic PCs, latitude, longitude and elevation of each sampled population.

**Table S1** Description of the 52 natural populations of Larix occidentalis and their phenotypes

**Table S2** Regression coefficients (R2) between phenotypic traits and geographic and climatic variables of the source location for each natural population.

**Figure S1.** Drought experiment timeline showing when rain covers were put over the raised bed (grey), when water was excluded (yellow) and when treatments were regularly watered (blue). Phenotypic traits as symbols show rough timing of measurements in 2021 and 2022 (drought and recovery season, respectively).

**Figure S2.** Annual growth and bud set phenology for the control, moderate, and severe drought treatments in western larch seedlings in the drought year (2021). Bold points represent the BLUEs for each treatment and lighter points represent individual data. Letters indicate statistically significant differences between groups (Tukey’s HSD test, α = 0.05).

**Figure S3.** Fluorescence (Fv/Fm) values and decline curves over time in the control and severe drought treatment. Points and lines represent natural population phenotypes (BLUPs) and red-orange color gradient represent fluorescence decline (slope) in the severe drought treatment. Fluorescence values in the control treatment are shown in green.

**Figure S4.** Proportion of mortality in the severe drought treatment by each natural population (seedlot). Orange bars represent proportion of seedlings that were dead by the end of the drought treatment and green represents proportion of seedlings that survived. Black line represents the average for all populations.

**Figure S5.** Canopy loss (%) in the moderate and severe drought treatment for natural populations. Bold points represent BLUEs of each treatment and lighter points represent individual data. Letters indicate statistically significant differences between groups (Tukey’s HSD test, α = 0.05).

**Figure S6.** Early height growth (cm) by treatment in the recovery year (2022). Bold points represent BLUEs for each treatment and lighter points represent individual data. Letters indicate statistically significant differences between groups (Tukey’s HSD test, α = 0.05).

**Figure S7.** Results of the partial RDA showing the loadings of each locus (SNPs; grey points), population allele frequencies (green points), and the predictor variables along the first two RDA axes (left) and the first and third axes (right). The locus scores (grey points) are rescaled to an unshown axis for better visibility.

**Figure S8.** Pearson correlations between pRDA predictor variables including the five selected climate variables, five neutral genetic PCs, latitude, longitude and elevation of each sampled population.
